# Machine learning discovery of cost-efficient dry cooler designs for concentrated solar power plants

**DOI:** 10.1038/s41598-024-67346-6

**Published:** 2024-08-17

**Authors:** Hansley Narasiah, Ouail Kitouni, Andrea Scorsoglio, Bernd K. Sturdza, Shawn Hatcher, Kelsi Katcher, Javad Khalesi, Dolores Garcia, Matt J. Kusner

**Affiliations:** 1 University College London, London, UK; 2 Massachusetts Institute of Technology, Cambridge, USA; 3 University of Arizona, Tucson, USA; 4 University of Oxford, Oxford, UK; 5 Mississippi State University, Starkville, USA; 6 Southwest Research Institute, San Antonio, USA; 7 UNC Charlotte, Charlotte, USA; 8 CERN, Geneva, Switzerland

**Keywords:** Dry cooling, Concentrated solar power, Bayesian optimization, Inverse design, Solar thermal energy, Energy science and technology, Renewable energy

## Abstract

Concentrated solar power (CSP) is one of the few sustainable energy technologies that offers day-to-night energy storage. Recent development of the supercritical carbon dioxide (sCO2) Brayton cycle has made CSP a potentially cost-competitive energy source. However, as CSP plants are most efficient in desert regions, where there is high solar irradiance and low land cost, careful design of a dry cooling system is crucial to make CSP practical. In this work, we present a machine learning system to optimize the factory design and configuration of a dry cooling system for an sCO2 Brayton cycle CSP plant. For this, we develop a physics-based simulation of the cooling properties of an air-cooled heat exchanger. The simulator is able to construct a dry cooling system satisfying a wide variety of power cycle requirements (e.g., 10–100 MW) for any surface air temperature. Using this simulator, we leverage recent results in high-dimensional Bayesian optimization to optimize dry cooler designs that minimize lifetime cost for a given location, reducing this cost by 67% compared to recently proposed designs. Our simulation and optimization framework can increase the development pace of economically-viable sustainable energy generation systems.

## Introduction

**Figure 1:**
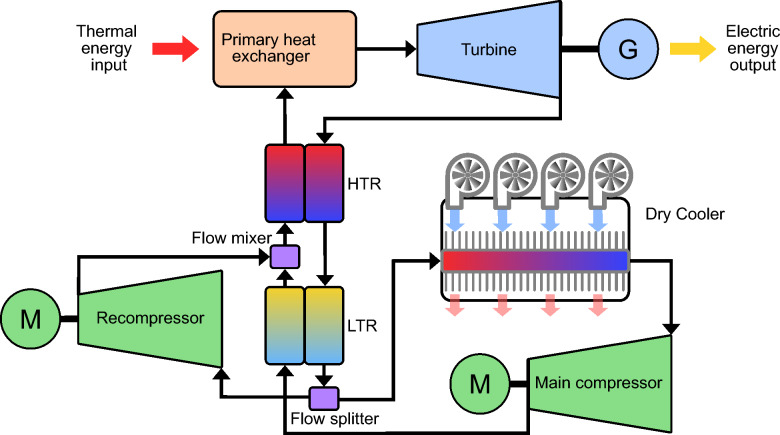
Schematic of a dry cooled sCO$_2$ **CSP plant.** The high temperature and low temperature recuperators are labeled with HTR and LTR, respectively.

**Figure 2:**
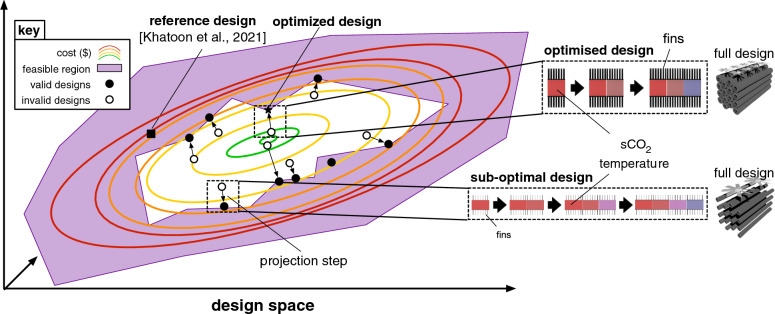
The optimization process. In each optimization step our algorithm starts by selecting a dry cooler design (shown in circles). If the initial design does not satisfy constraints on temperature and pressure, it lengthens the cooler tubes until the constraints are satisfied. This can be seen as a projection step onto the feasible region of valid dry coolers. Given this, we obtain a design cost (shown by the colored contour lines). We use this cost to update a Gaussian process estimate of the global cost, and then use Bayesian optimization to select a new design to minimize the overall cost. This processes is repeated, continually reducing design cost.

There is scientific consensus that increasing greenhouse gas concentrations in the atmosphere lead to warmer average temperatures across the globe^1^. These higher temperatures have increased extreme weather events^1^, and the United Nations have issued a statement claiming that $90\%$ of all disasters are related to this increase^2^. The majority source of these greenhouse gases is fossil fuels for energy (for example, roughly $80\%$ of all emissions for the USA and for the European Union)^3^. As such, decarbonizing energy production is critical to reducing the extreme effects of global warming^1^. Sustainable energy needs to generate greater than $60\%$ of all energy by 2050 to meet the warming target set by the Paris Agreement^1^. Reducing the cost of current sustainable energy generation technologies will increase the chances of meeting such targets. One renewable energy technology that has recently experienced dramatic reductions in cost is concentrated solar power (CSP): a $68\%$ reduction in levelized cost of electricity (LCOE) from 2010 to 2020^4^. A number of factors have contributed to this decrease, including reduced installation costs and increased energy storage times. CSP is one of the few sustainable energy generation technologies equipped with reliable, low-cost energy storage, via stores of heated working fluid^5^. Recently, preliminary results on a new Brayton power cycle for CSP based on supercritical carbon dioxide (sCO$_2$) have promised the ability to further reduce material costs and improve reliability at extreme temperatures^6,7^. However, the design of sCO$_2$ Brayton cycle CSP plants is still preliminary, see (Fig. 1 Fig. 2^8^).

One crucial aspect of sCO$_2$ CSP plant design is the cooling system. This is due to CSP plants having the highest energy potential in desert regions^9,10^ which makes traditional water-based coolers not economically feasible^11^. To address this, recent work has investigated dry cooling as a possible solution^12^. However, because the use of sCO$_2$ recompression Brayton cycles for CSP is still in its infancy, there has only been limited investigation into this^12–14^. Specifically, prior work focuses mainly on the effect of varying power cycle set points and ambient conditions^13,14^, and largely omit varying the sCO$_2$ CSP dry cooler design. In fact, only one study by Ehsan et al.,^15^ varies individual dry cooler design parameters, see Table 1 for more details .

**Table 1:** Comparison of varied parameters for prior art and this work.

Varied Parameters	Khatoon^13^	Wang^14^	Ehsan^15^	This work
Ambient conditions	✗	✓	✓	✓
Power cycle set points	✓	✓	✓	✓
Dry cooler parts (separately)	✗	✗	✓	✓
Dry cooler parts (all, jointly)	✗	✗	✗	✓

This is because cooler design is complex: the thermal performance of the sCO$_2$ recompression cycle is highly dependent on the main compressor inlet temperature, which is controlled by the cooler^15^. However, many design factors contribute to the effect of the cooler on this temperature (tube and fin diameters, pitches, thicknesses, fan configurations, etc...), few of which have been changed. For this reason, current dry cooler designs are far from optimal. To bring the cost of renewable energy down, in this paper we propose a machine learning system to automate the cost-efficient design of dry cooling for CSP plants. Our system is composed of a high-fidelity simulator of a compact cross-flow finned-tube heat exchanger, combined with a powerful high-dimensional Bayesian optimization method. Specifically, we simulate the heat transfer between hot supercritical CO$_2$ flowing through tubes and a colder air mass, which is forced across fins on the outside of the tubes via fans. An element-wise calculation of the heat transfer between sCO$_2$ and air for a given design is propagated through the entire heat exchanger via the logarithmic mean temperature difference (LMTD) method (see Methods for details). This calculation is used to automatically adjust the length of finned tubes until an output temperature is reached that ensures supercriticality is maintained. In this way, the simulator ensures all physical constraints on the heat exchanger are met. This enables us to integrate a recent trust region Bayesian optimization method, TuRBO^16^ to minimize design cost. Specifically, the outputs of our heat exchanger simulator are processed by the optimizer, which suggests a new design configuration for the next iteration of the simulator. This process is repeated until a minimum cost design is found. The proposed pipeline is described in Fig. 1. This inverse-design approach improves upon prior work^12,13,15,17^, by optimizing all tube and fin dry cooler parameters *simultaneously*, whereas prior work only investigates changing one design parameter at a time^15^. Our approach is transferable from CSP plants to other applications of air cooling and thus holds tremendous potential to increase efficiency and reduce the cost of air cooling in any scenario.

## Results

### Simulating an sCO$_2$-based cross-flow dry cooler

**Figure 3:**
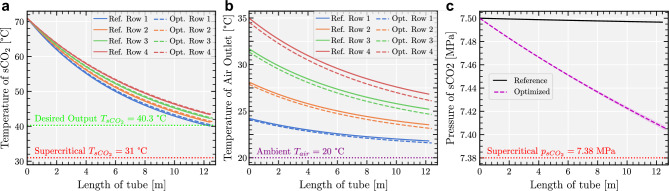
Simulated parameter variations along the length of the tube and across the rows of the heat exchanger. (**a**) Temperature variation of sCO$_2$. (**b**) Outlet air temperature variation. (**c**) Mean pressure (dashed) and min-max pressure (shaded) variations of sCO$_2$.

Here we propose a dry cooler simulator that calculates the heat transfer between sCO$_2$ working fluid in finned tubes and cross-flowing air. To do so we use the well-established Logarithmic Mean Temperature Difference (LMTD) method^18–20^ to element-wise propagate the heat transfer along the finned tubes. The simulator uses this to compute an overall Heat Transfer Coefficient (HTC) based on tube geometry, fluid properties, and flow conditions. It additionally calculates the pressure drop across each tube segment using correlations, such as the Darcy-Weisbach equation^21^. The direct calculation of the outgoing thermodynamic properties in the heat exchanger is complicated due to the complex, non-linear, and non-differentiable factors involved in computing the overall heat transfer coefficient within each segment. Therefore, an iterative approach involving a binary search is used to solve for the outgoing $\text {sCO}_2$ temperature. This strategy is based on the assumption that the overall heat transfer coefficient, $O_{{\textrm{htc}}_{(i,j)}}$, and by extension the heat transferred, is a monotonic function of outgoing $\text {sCO}_2$ temperature^13,22^. It is possible that, for a set of design parameters, the simulation run does not achieve the required outlet $\text {sCO}_2$ properties. To guarantee this, the length of the tubes is dynamically adjusted to ensure the required $\text {sCO}_2$ properties are met. This design flexibility is visually represented in Fig. 3a, which showcases the relationship between tube length and output temperature of the sCO$_2$. This has the effect of producing designs for which the output sCO$_2$ output temperature is always satisfied, i.e. the framework produces only valid designs as per our imposed requirements—in this case the output sCO$_2$ temperature.

Figure 3 shows that the supercritical $\text {CO}_2$ conditions, temperature above $31^\circ$C and pressure above 7.38 MPa, are maintained across all design variations. Variations in temperature and pressure along the tube lengths and across the rows of the heat exchanger are carefully monitored to ensure operational efficiency and integrity.

Figure 3c shows a significant difference between the pressure drops between the reference and the optimized designs due to the difference in the inner diameter of the two designs. Indeed, the pressure drop has an inverse relationship with the inner tube diameter of the designs, as per equation 30 in Supplementary Text S4 (i.e. when the diameter of inner tube is decreased, the pressure drop increases and vice-versa). Moreover, the pressure drop gradually decreases along the length of the finned-tubes due to the decreasing temperature drop of the $\text {sCO}_2$ along the same length in turn explaining the “flattening” of the pressure drop towards the end of the finned-tubes. The simulation is based in classical energy conservation principles, used in prior work^13,15,17^ and described in more detail in Supplementary Text S4. All the model parameters and design bounds are provided in Supplementary Text S6.

Given a design we calculate the cost of the resulting dry cooler, considering material, operational, and maintenance expenses, inspired by related work^12,23,24^. By integrating real-world pricing data and operational metrics, we derive an estimate of the lifetime cost of the dry cooler. More details about the implementation of this is provided in the Methods and Supplementary Text S3.

### Automating dry cooler design anywhere in the world

Given a simulator of the physical properties and the cost of a dry cooler used in a Brayton cycle $\text {sCO}_2$ CSP plant, we show how to optimize its design to minimize its cost for any location in the world. Traditional optimization techniques based in mathematical programming or gradient descent cannot be directly applied: the simulator cannot be written in standard form as it relies on calls to external libraries including CoolProp^25^, to fetch temperature and pressure data. Further, since the run time to simulate a physically-valid dry cooler design is substantial (see Supplementary Text S5 for timing details), we need an optimization method that carefully selects new designs to minimize the total number of designs searched.

To satisfy these constraints we leverage recent work in high-dimensional Bayesian optimization^16^. Bayesian optimization is a global optimization method that allows for a principled trade-off between exploration and exploitation^26^. It uses a surrogate model, often a Gaussian process (GP)^27^, to model the expensive objective function, taking into account uncertainty. Recent work has shown that for high-dimensional problems, sample efficiency and scalability improves when the surrogate models are split into local models^16^. This enables us to automatically search a vast space of valid dry cooler designs to find a low-cost solution.

**Figure 4:**
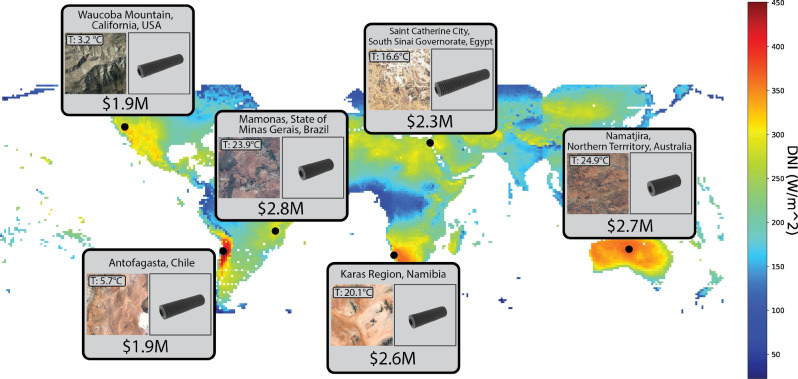
Comparison of optimized air cooler cost at selected locations on Earth. The color scale indicates the mean direct normal irradiance (DNI) across the globe, as per NREL-NSRDB^28^. A high DNI was used as a precondition in CSP plant site selection (full details in Supplementary Text S2). The air cooler is optimized for a 25 MW CSP plant.

We use the National Renewable Energy Laboratory’s National Solar Radiation Database (NREL-NSRDB)^28^ to obtain yearly mean temperature and Direct Normal Irradiance (DNI) data at single-degree intervals. We initially select the 800 locations with the highest mean yearly DNI. We filter these locations further by identifying all groups of points within contiguous geographic regions and selecting the location within each region that maximizes the DNI value. This results in the final 6 locations that we use in the experiment. Full details of the data curation process are described in Supplementary Text S2.

The selected locations span arid deserts and humid tropics, allowing us to test the versatility and adaptability of our proposed framework to different climates. For each location, we use our framework to automatically design a sCO$_2$ CSP dry cooler that minimizes lifetime cost (reported in USD). The results are shown in Fig. 4.

As expected, designs found by our framework have the lowest cost in locations with higher mean DNIs and lower ambient temperatures, i.e., in Waucoba Mountain, California, USA (mean temperature: 3.2°C). and in Antofagasta, Chile (mean temperature: 5.7°C). In these locations the overall tube lengths can be shorter as the cooler air more effectively cools the sCO$_2$ working fluid than in hotter environments.

**Figure 5:**
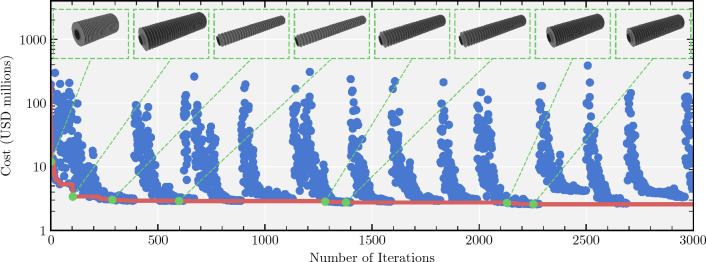
Optimization curve. Each blue dot represents one iteration of the simulator and corresponds to a valid design. The red line shows the current minimum cost found across all designs searched to that point. The green points illustrate the tube designs of minimum-cost designs at different stages in the optimization process.

For a single location we visualize a single optimization run in Fig. 5. The blue points show the cost of all designs that obtained higher cost than the best design found at the time, highlighting the difficulty of the design optimization problem. The red line highlights the cost of the lowest-cost design found so far. We observe that the optimizer is able to quickly reduce the cost in the first 100 iterations. After this point it fine tunes the design to continue to lower the cost. At the top of the figure we display the evolution of new lowest-cost designs during the optimization process. These designs are lower in cost than all previous designs, while maintaining supercriticality of the working fluid, and the desired output temperature.

One key aspect of dry cooler design is the ambient air temperature. In a sCO$_2$ CSP plant, where the working fluid must be cooled to temperatures near its critical point at $31^{\circ }$C (our design has a set point of $40.3^{\circ }$C), achieving adequate cooling becomes challenging as ambient temperatures rise. For effective cooling, either the surface area dedicated to heat exchange, or the airflow, or both, must be increased. Both adjustments affect the lifetime cost of the air cooler in a non-linear fashion, making the identification of an optimal solution challenging.

**Figure 6:**
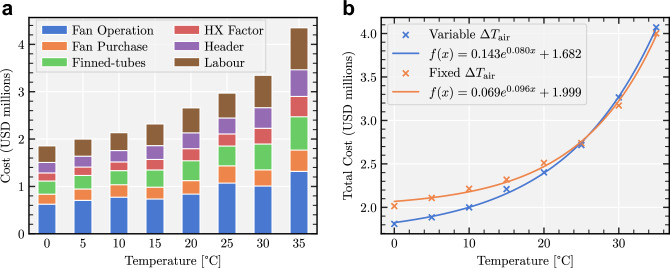
Comparison of optimized air cooler cost for a 25 MW CSP plant at different ambient temperatures. (**a**) Breakdown of the minimum optimized cost by air cooler components for variable $\Delta T_{\textrm{air}}$ (blue data on right) shown for different ambient temperatures. (**b**) Minimum total lifetime cost of the air cooler at different ambient temperatures. The lifetime cost follows exponential fits, the larger deviation from the fit in the blue data is a result of the larger parameter space.

Our algorithm allows one to generate optimized air cooler designs for any given ambient air temperature. We evaluate the temperature sensitivity studies of our approach in two ways:

1. $\Delta T_{\textrm{air}} = T_{{\textrm{air}}_{\textrm{out}}} - T_{{\textrm{air}}_{\textrm{in}}}$, is kept constant: This is the less complex scenario of the two. The temperature difference between incoming and outgoing air, $\Delta T_{\textrm{air}}$ is kept constant regardless of the ambient air temperature. This implies that the airflow remains constant, and only the heat exchanger’s surface area changes with varying temperature, thereby restricting the heat exchanger’s effectiveness to predefined values. In this scenario, the algorithm optimizes the heat exchanger’s design and calculates the optimized lifetime cost (red data in Fig. 6b).
2. $\Delta T_{\textrm{air}}$ is a variable: in this scenario, both the surface area and the air flow are simultaneously adjusted. The results (blue data in Fig. 6b) demonstrate that this broader approach/scenario allows for an additional reduction in the lifetime cost of the air-cooler, as expected since this scenario, unlike the first one, does not force the design to be able to transfer a specific amount of energy from the $\text {sCO}_2$ to the incoming air at ambient temperature. This additional cost reduction is reduced significantly at higher ambient air temperatures due to the parameter space getting smaller.

Figure 6 shows that both datasets follow an exponential fit in their minimum costs as temperature varies. The difference in “constant $\Delta T_{\textrm{air}}$” and “variable $\Delta T_{\textrm{air}}$” minimum optimized costs in the figure can be explained by the fact that the range of temperatures that the output air temperature can take and that are consistent with energy conservation principles gets bigger with decreasing ambient air temperatures. In other words, imposing a constant $\Delta T_{\textrm{air}}$ restricts the framework’s optimization capabilities in this parameter space, thereby limiting the potential savings in the lifetime cost of the optimized design, and since this space increases with decreasing temperatures, the difference in costs between the two scenarios also increases with decreasing temperatures. The components of the lifetime costs in the second scenario are shown in Fig. 6a. The results show that both the heat exchanger cost and the cost of the fans increase with temperature. The variations in fan power cost stem from the algorithm’s selection of different fan types based on varying ambient temperatures.

This underscores the significance of temperature sensitivity in air cooler design, emphasizing the need for adaptive and flexible design strategies to ensure consistent performance in fluctuating temperatures. We hope this work paves the way for harnessing solar energy potentials in a multitude of regions, accelerating the transition to sustainable energy solutions.

### Improvements over prior work

We compare our approach to the recent work of Khatoon *et al.*^13^ who design a dry cooler for a specific ambient temperature for a $25\,$MW CSP plant. In contrast to our results, their design choices are based on previous work by Ehsan *et al.*^17^. Table 2 shows how our design ‘Optimized Value’ compares to the previous design ‘Reference Value’.

**Table 2:** Comparison of parameters for the referenced design and optimized designs. All values are expressed in millimeters.

Design parameter	Optimized	Reference^13^
Tube inner diameter, $d_{\textrm{tube}}^{\textrm{in}}$	10.452	20.000
Tube outer diameter, $d_{\textrm{tube}}^{\textrm{out}}$	11.497	25.000
Fin inner diameter, $d_{\textrm{fin}}^{\textrm{in}}$	11.673	28.000
Fin outer diameter, $d_{\textrm{fin}}^{\textrm{out}}$	22.962	57.000
Tube transverse pitch, $S_{\textrm{T}}$	37.558	58.000
Fin pitch, *s*	2.827	2.800
Fin thickness, $t_{\textrm{fin}}$	0.286	0.500

**Figure 7:**
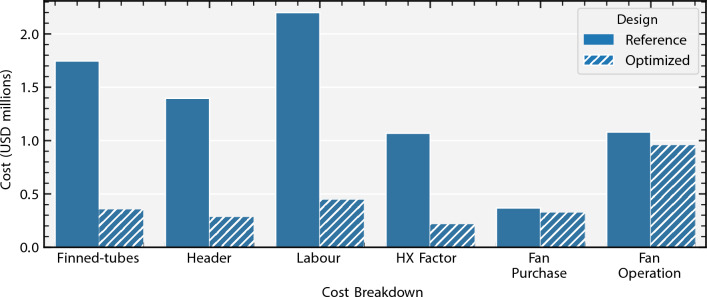
Lifetime cost breakdown comparison between reference design and fully-optimized design. Parameter values as per Table 2. Note that the biggest savings come from finned-tube and labor costs, while the smallest reduction comes from fan-related costs.

**Figure 8:**
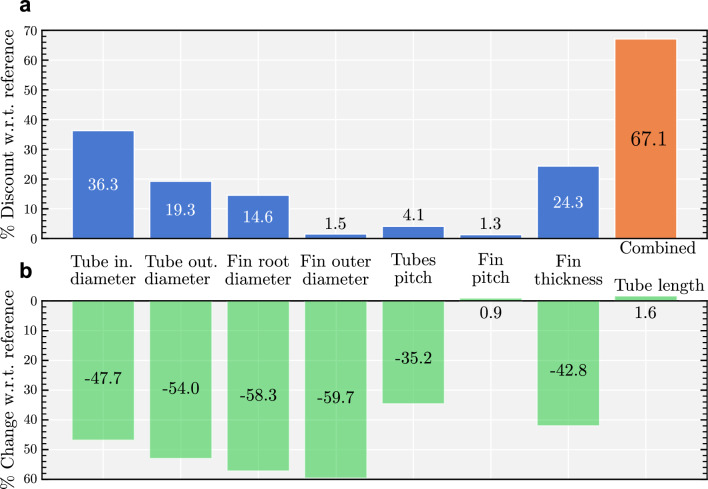
Cost sensitivity of different parameters and summary of the differences between the reference and the fully optimized designs as per Table 2. (**a**) Cost sensitivity of each parameter in terms of percentage discount w.r.t. reference design. This is calculated by changing one parameter at a time to match the optimized value found by the optimization process while keeping the others fixed to their original reference values, as in Table 2. The difference in cost is then computed with respect to the reference design and presented in this figure. (**b**) Relative change between the optimized and reference design parameters.

Figure 7 breaks down the cost categories for each of the designs. The largest reductions in cost come from finned-tube and labor costs, while the smallest reduction comes from fan-related costs. Note that the former are a result of the savings from material quantities (see Supplementary Text S3).

Figure 8 shows the change in cost and amount of material used in tube construction between the reference design^13^ and our optimized design. Figure 8a shows that the optimized design lowers the cost tube inner diameter by more than $35\%$ and the tube outer diameter by almost $20\%$. It does so by shrinking the overall size of the tube by $54\%$, and thinning the overall tube, as shown in Fig. 8b. Overall our optimized design is a 67.1% reduction in the lifetime cost of the dry cooler from the reference design (Fig. 8a).

## Discussion

To reduce the damaging effects of climate change, an increase in sustainable energy generation is needed. This increase can be accelerated if the cost of sustainable energy technologies is reduced. One such technology that is especially promising is concentrated solar power (CSP), one of the few sustainable energy generation systems with cheap, reliable energy storage. Here, we present a system for automatically designing a key component of an sCO$_2$ CSP plant, the dry cooler, in order to minimize construction and operation cost. Compared to existing design approaches, our system discovered a dry cooler that is 67% cheaper that still keeps the working fluid in the required supercritical state. This system can be used to find a low cost cooler design customized to any location on earth.

The system itself is modular, in the future one can customize the dry cooler simulator and the cost calculation as materials and production changes. One can also tailor the physical modeling to suit different working fluids and cooling systems (e.g., cooling towers). In principle, our approach can be extended to the design of the full CSP plant. This could be done by simulating other components of the plant, and their impact on power generation and lifetime cost. This work suggests that future work on physical simulation of sustainable energy generation, as well as black-box optimization techniques such as Bayesian optimization, can help reduce the cost of these technologies.

It is important to note that the current design of optimum parameters for the dry cooler does not account for uncertainties in various design parameters, such as solar irradiance, operating of sCO$_2$ temperatures and pressures entering the cooling system. These uncertainties can impact system performance and output.

The code and the framework we have developed does allow for adjusting the design points of the cooling cycle output, such as the temperature and pressure of the sCO$_2$ entering the cooling system. While we did not incorporate these adjustments in our current study for simplicity, we recognize that this is a useful direction for future work.

Considering a range of ambient air temperatures at each CSP plant location, rather than using the mean temperature, could help produce lower-cost operating designs tailored to specific locations. This is because the optimum design parameters will ultimately depend on the environmental conditions and their variations at the proposed locations, and integrating these considerations would ensure more robust and adaptive performance across these varying conditions, thereby reducing cost while maintaining necessary conditions of the power cycle across time.

## Methods

### Optimization overview

In this section, we give an overview of the optimization problem and our approach. Note that the notations in this section are unique to this section of the report. The methodological approach is framed around a well-defined optimization goal:

1 $$\begin{aligned} \text {minimize } c({\textbf{x}}) \text { such that } v({\textbf{x}}) = 1 \end{aligned}$$

Within this construct, ${\textbf{x}}$ represents a design configuration of the dry cooler. The cost function $c({\textbf{x}})$ calculates the cost associated with this design (also referred to as the cost calculator). Meanwhile, $v({\textbf{x}})$ is a binary function designed to assess the viability of the design. When $v({\textbf{x}})$ outputs a value of 1, it implies that the design meets the desired output temperature criteria while preserving the supercritical state of the CO$_2$ throughout.

At its core, this optimization task is a complex non-linear mixed-integer programming problem, as the design vector ${\textbf{x}}$ contains both continuous and integer variables, and both the cost function $c({\textbf{x}})$ and the validity function $v({\textbf{x}})$ have non-linear relationships with ${\textbf{x}}$. Solving such a problem using traditional mixed-integer programming techniques would be computationally daunting and most likely intractable. Instead, we propose to reformulate the problem into an equivalent one:

2 $$\begin{aligned} \text {minimize } c(p({\textbf{x}})) \end{aligned}$$

Here, $p({\textbf{x}})$ refers to a projection function (also referred to as the simulator). Given a design vector ${\textbf{x}}$, it returns a modified design vector $\mathbf {x'}$ such that $v(\mathbf {x'}) = 1$. This function ensures that the output design adheres to the critical criteria, namely, achieving the target temperature while maintaining the CO$_2$ in its supercritical state.

### Bayesian optimization

While much of modern machine learning uses gradient-based methods to solve optimization problems^29^, there are many cases, such as Eq. (2), where gradients are intractable to compute and/or do not exist. In this case, one approach is to approximate the unknown objective function using a surrogate function^30^. One class of highly-successful surrogate-based optimization techniques is Bayesian optimization^31^. Given this surrogate, the question we need to answer is how to select new designs ${\textbf{x}}$ that will minimize Eq. (2). Crucially, this selection must balance *exploration*: designs that are very different from previously-seen designs may have very low cost values, and *exploitation*: slightly tweaking existing designs may give large reductions in cost. Bayesian optimization balances exploration and exploitation by using a surrogate function that includes an estimation of uncertainty (e.g., often this is via a Gaussian process^31^).

TuRBO^16^ is a recently-proposed Bayesian optimization method that improves scalability to high-dimensional inputs, such as the complex collection of design parameters that make up a dry cooler in sCO$_2$ CSP plants. It improves scalability by replacing the inefficient and imprecise global surrogate model of traditional Bayesian optimization with several independent local surrogate models. Each local surrogate model is a Gaussian process (GP) that is updated with its own set of samples, allocated by a multi-armed bandit algorithm. Each model is located within a trust region (TR), a hyperrectangle centered at the current best local solution. As new samples are seen not only might the location of the TR change, but also the shape, as each side is scaled according to the corresponding lengthscale in the GP model. Further, inspired by the Nelder-Mead algorithm^32^, the overall size of the TR is expanded if there are too many consecutive points that improve upon the best point so far, and it is shrunk if there are too many points that do not improve the best solution. Given a set of *m* local TRs, with corresponding independent GP models, TuRBO balances the exploration/exploitation trade-off using Thompson Sampling^33^. Specifically, to select the next design ${\textbf{x}}_i$ across all TRs TuRBO first draws a posterior function from each local GP in each TR: $f_\ell ^{(t)}({\textbf{x}}) \sim \mathcal{G}\mathcal{P}_\ell ^{(t)}(\mu _\ell ({\textbf{x}}), k_\ell ({\textbf{x}}, {\textbf{x}}'))$, where $\ell$ indexes the trust region, and *t* is the iteration of Bayesian optimization. Given these it then selects the design that is minimal across all of these functions:

$$\begin{aligned} {\textbf{x}}_i \in \mathop {\mathrm {arg\!\,min}}\limits _\ell \mathop {\mathrm {arg\!\,min}}\limits _{{\textbf{x}} \in \textrm{TR}_\ell } f_\ell ^{(t)}({\textbf{x}}), \;\; \textrm{where} \;\; f_\ell ^{(t)}({\textbf{x}}) \sim \mathcal{G}\mathcal{P}_\ell ^{(t)}(\mu _\ell ({\textbf{x}}), k_\ell ({\textbf{x}}, {\textbf{x}}')). \end{aligned}$$

Optimizing across these sampled posterior functions $f_\ell ^{(t)}$ allows TuRBO to incorporate the uncertainties modeled by the Gaussian processes $\mathcal{G}\mathcal{P}_\ell ^{(t)}$: if a GP model has high variance, the sampled function will vary each iteration. If the sampled function has very small values, Bayesian optimization is more likely to explore the corresponding trust region, whereas if it has high values it is more likely to explore/exploit other trust regions.

In our experiments, we use 5 trust regions in all the experiments presented in this paper. We note that the overall setup of Bayesian optimization is different than standard machine learning: there is no initial training dataset, but data is instead collected over time by sampling designs ${\textbf{x}}$ every optimization iteration, as in Fig. 5. The set of design parameters ${\textbf{x}}$ that are optimized are detailed in Table 2. We run optimization until 3000 iterations which appears to be large enough as the minimum cost stabilizes as shown by the red line in Fig. 5. Overfitting is luckily not an issue for Bayesian optimization as, in general, we would like to fit the true objective as well as possible.

TuRBO^16^ was compared with multiple state-of-the-art (SOTA) methods for high-dimensional black-box optimization including other Bayesian optimization methods: BOHAMIANN^34^ which replaces the usual Gaussian process surrogate with a neural network and uses Hamiltonian Monte Carlo to calculate uncertainties, BOCK^35^ which transforms the search space using a cylindrical mapping to improve scalability, EBO^36^ that is an ensemble of additive GPs, and HeSBO^37^ the uses a novel subspace embedding to overcome limitations of previous Gaussian projections. The comparison also included non-Bayesian optimization methods: BOBYQA^38^, a trust-region approach that uses a quadratic approximation of the objective, the Nelder-Mead algorithm^32^, which creates a simplex that adaptively moves along the surface of the true objective function, and BFGS^39^, a quasi-Newton method that approximates gradients using finite differences. The comparison included a diverse range of complex and multi-modal problems, which are challenging for many global optimization algorithms. These problems included a 14-dimensional (14D) robot pushing problem, a 60-dimensional (60D) rover trajectory planning problem, a 12-dimensional (12D) cosmological constant estimation problem, a 12-dimensional (12D) lunar landing reinforcement learning problem, and a 200-dimensional (200D) synthetic problem^16^. The comprehensive experimental evaluation demonstrated that TuRBO^16^ outperforms these state-of-the-art Bayesian optimization and non-Bayesian optimization methods across various real-world complex tasks, underscoring its robustness and effectiveness in tackling high-dimensional optimization challenges, such as the one presented in this paper.

### Simulator implementation

The heat exchanger simulator is designed to model and analyze the performance of air-cooled sCO$_2$ coolers, taking into account various design parameters such as tube dimensions, fin properties, and flow conditions. The implementation employs a combination of energy conservation and empirical correlations to simulate the heat transfer process between CO$_2$ and air streams.

Several assumptions are made in the simulator to simplify the analysis and we provide a detailed account of how this was implemented in Supplementary Text S4. These include steady-state operation with constant mass flow rates for both CO$_2$ and air, constant thermophysical properties within each segment, uniform air distribution across tubes, negligible pressure drop for air, and a segmented approach where tubes are divided into multiple segments treated as individual heat exchanger units.

Because sCO2 CSP heat exchanger development is still in its infancy, we do not have access to physical measurements and are unaware of any that are publicly available. However, our simulator equations are based in prior works^17^ which have been validated against well-studied textbook models^23^. For additional details see Supplementary Text S4.

### Cost calculator implementation

Accurately modeling the manufacturing and operational costs is crucial for optimization. We implement a cost calculator based on prior work^12,23,24^ but refine and modularize it for this application. The model has three main components: **(1)** Heat exchanger cost: Accounts for materials, labor, overheads, and other factors. **(2)** Fan purchase cost: Initial outlay based on required air flow rates. **(3)** Fan operation cost: Electricity usage over lifetime from power ratings. (see Supplementary Text S3 for details).

### Supplementary Information

Supplementary Information.

## Data Availability

The simulation results used and analysed during this study are available from the corresponding author on reasonable request. The entire simulator code for this project is on gitlab, see Supplementary Text S1.

## Code availability

See Supplementary Text S1.
