# Supplementary material for: Machine learning discovery of cost-efficient dry cooler designs for concentrated solar power plants
Source: Sci Rep. 2024 Aug 17;14:19086. doi: 10.1038/s41598-024-67346-6 (PMC11330457; doi:10.1038/s41598-024-67346-6)
Supplement: Supplementary file 1 — Supplementary Information. [file 41598_2024_67346_MOESM1_ESM.pdf]

Supporting Information: Machine Learning Discovery of Cost-Efficient Dry Cooler Designs for Concentrated Solar Power Plants

Hansley Narasiah,[†,△] Ouail Kitouni,[‡,△] Andrea Scorsoglio,[¶,△] Bernd K. Sturdza,[*,§,△] Shawn Hatcher,[‖] Kelsi Katcher,[⊥] Javad Khalesi,[#] Dolores Garcia,[@] and Matt J. Kusner[*,†]

†*University College London, UK*

‡*Massachusetts Institute of Technology, USA*

¶*University of Arizona, USA*

§*University of Oxford, UK*

‖*Mississippi State University, USA*

⊥*Southwest Research Institute, USA*

#*UNC Charlotte, USA*

@*CERN, Switzerland*

△*Contributed equally to this work*

E-mail: bernd.sturdza@physics.ox.ac.uk; m.kusner@ucl.ac.uk

Supplementary Text S1 Source Code

Source code for all of the methods implemented in this project can be found in the GitLab repository: `https://gitlab.com/frontierdevelopmentlab/2022-us-cspcontroller`. The code for this project originated from the Frontier Development Lab 2022 challenge and is built upon and pushed into the repository above. The code repository is divided into four parts:

- LMTD simulator: contains all the code relating to the simulators described in Supplementary Text S4.
- NREL Data: contains all code relating to the NREL data extraction and processing as described in Supplementary Text S2.
- CFD: contains all the code used in the attempt to replace the current simulator with a multi-physics simulator using OpenFOAM.[S1] Not presented in this paper.
- gun-unet: unused in this project.

Supplementary Text S2 Location-Specific Optimization

Provided that local weather data is available, the methodology used in this report can optimize the design of the dry-cooler for any chosen location worldwide. To illustrate this capability, temperature and Direct Normal Irradiance (DNI) data obtained from the National Renewable Energy Laboratory's National Solar Radiation Database (NREL-NSRDB)[S2] at 1-degree intervals was used to evaluate the tool's performance. The following steps were taken in the preparation of this data and the code is included in Supplementary Text S1:

1. Raw data files are extracted via the NREL-NSRDB API at each location on the globe given by their latitude and longitude. The locations are selected at 1-degree intervals in this global coordinate system. This results in a list of time-series databases (stored

as CSV[S3] files) for each location containing 60-minute interval data, which includes the entries relevant for the simulator, i.e. DNI and air temperature, as well as other entries such as wind speed and solar zenith angle amongst others. Note that during this step, locations that are not on land won't return any data and instead return errors for those locations (this is not a bug in the URL build-up for the API call but a feature of the NREL-NSRDB[S2]).

2. Each of the above mentioned CSV files are then processed to find the mean DNI and mean air temperature across the entire year at each location. The result is two CSV files, one containing locations with their corresponding mean DNI data and the other containing locations with their corresponding mean air temperature data.

3. The resulting mean DNI data is sorted and the 800 locations with the highest mean DNI values are extracted.

4. The sites with the maximum DNI (of those pre-selected 800 locations from the previous step) within each continuous cluster of points are selected. The mean DNI and air temperature data for these locations are then stored in a separated CSV file. These are the sites which will be used as part of the optimization procedure.

The mean air temperature for the selected sites was then incorporated into the optimization algorithm, calculating the most efficient, cost-effective design for each of them. The results are presented in Figure 3.Note that in this figure, the optimization was run only once with 3000 iterations for each location.

Supplementary Text S3 Cost Calculations

The cost calculations are based on prior work.[S4–S6] Note the following assumptions in the calculation:

- All parts last an entire lifespan of 25 years.

- Cost of electricity in fan power consumption is assumed to be \$0.05 per kWh.
- No maintenance costs/fouling are considered.
- Fan loading is in an optimal range.
- Forced/induced draft is not considered separately.

The calculation procedure used to calculate the cost of the heat exchanger, that associated with the fan(s), and the resultant total lifetime cost of the heat exchanger, respectively are provided below:

$$L = L_{\text{segment}} \cdot n_{\text{segments}} \tag{S1}$$

$$\frac{c_{\text{tube}}}{L} = \frac{\pi \left({d_{\text{out}}^{\text{tube}}}^2 - {d_{\text{in}}^{\text{tube}}}^2 \right)}{4} \rho_{\text{t.m.}} c_{\text{t.m.}} \tag{S2}$$

$$\frac{c_{\text{fin}}}{L} = \frac{\pi}{4s} \rho_{\text{f.m.}} c_{\text{f.m.}} \left(\left({d_{\text{out}}^{\text{fin}}}^2 - {d_{\text{in}}^{\text{fin}}}^2 \right) t_{\text{fin}} + \left({d_{\text{in}}^{\text{fin}}}^2 - {d_{\text{out}}^{\text{tube}}}^2 \right) \cdot (s - t_{\text{fin}}) \right) \tag{S3}$$

$$\frac{c_{\text{finned-tube}}}{L} = \left(f_{\text{weighting}} \cdot \left(\frac{c_{\text{tube}}}{L} + \frac{c_{\text{fin}}}{L} \right) \right) + \frac{c_{\text{finned-tube}}^{\text{fixed}}}{L} \tag{S4}$$

$$c_{\text{finned-tubes}}^{\text{total}} = \frac{c_{\text{finned-tube}}}{L} \cdot L_{\text{segment}} \cdot n_{\text{segments}} \cdot n_{\text{tubes-in-row}} \cdot n_{\text{rows}} \cdot n_{\text{bundles}} \tag{S5}$$

$$c_{\text{air-cooler}}^{\text{no-fans}} = c_{\text{finned-tube}}^{\text{total}} \cdot (1 + f_{\text{header}}) \cdot (1 + f_{\text{labour}}) \cdot f_{\text{HX}} \tag{S6}$$

$$c_{\text{fans}}^{\text{initial}} = n_{\text{fans-required}} \cdot c_{\text{fan}}^{\text{purchase}} \tag{S7}$$

$$c_{\text{fans}}^{\text{operation}} = n_{\text{fans-required}} \frac{P_{\text{fan}}}{1000} \text{LCOE} \left(24 \cdot 365 \cdot n_{\text{lifetime-years}} \right) \tag{S8}$$

$$c_{\text{fans}}^{\text{lifetime}} = c_{\text{fans}}^{\text{initial}} + c_{\text{fans}}^{\text{operation}} \tag{S9}$$

$$c_{\text{HX}}^{\text{total}} = c_{\text{fans}}^{\text{lifetime}} + c_{\text{air-cooler}}^{\text{no-fans}} \tag{S10}$$

where:

- L: total length of the tubes,
- n_{segments}: number of segments comprising the length of tube,

- c_{tube}: cost of tube material in 1 finned-tube,
- $d_{\text{out}}^{\text{tube}}$: outside diameter of tubes,
- $d_{\text{in}}^{\text{tube}}$: inside diameter of tubes,
- $\rho_{\text{t.m.}}$: density of tube material [kg m^{-3}],
- $c_{\text{t.m.}}$: cost of tube material [\$ / kg],
- c_{fin}: cost of fin material in 1 finned-tube,
- s: fin pitch, $\rho_{\text{f.m.}}$: density of fin material [kg m^{-3}],
- $c_{\text{f.m.}}$: cost of fin material [\$ / kg],
- $d_{\text{out}}^{\text{fin}}$: outside diameter of fins,
- $d_{\text{in}}^{\text{fin}}$: inside diameter of fins,
- t_{fin}: thickness of fins,
- $c_{\text{finned-tube}}$: cost of producing 1 finned-tube,
- $f_{\text{weighting}}$: weighting factor of tube and fin material to estimate total material quantities,
- $c_{\text{finned-tube}}^{\text{fixed}}$: fixed costs associated with producing the finned-tubes,
- $c_{\text{finned-tubes}}^{\text{total}}$: total cost of producing all the finned-tubes in the heat exchanger,
- $n_{\text{tubes-in-row}}$: number of tubes in a row per bundle,
- n_{rows}: number of rows of finned-tubes in the heat exchanger,
- n_{bundles}: number of bundles in the heat exchanger,
- $c_{\text{air-cooler}}^{\text{no-fans}}$: cost of air cooler only (without any fan-associated costs),
- f_{header}: factor applied to calculate the header costs for producing heat exchanger,

- f_{labour}: factor applied to calculate the labour costs in producing the heat exchanger,
- f_{HX}: factor applied on top for entire heat exchanger,
- $c_{\text{fans}}^{\text{initial}}$: cost of purchasing all the fans required to compensate for air pressure drop,
- $n_{\text{fans-required}}$: number of fans required to compensate for air pressure drop,
- $c_{\text{fan}}^{\text{purchase}}$: cost of purchasing 1 fan,
- $c_{\text{fans}}^{\text{operation}}$: cost of operation of fans throughout their lifetime,
- P_{fan}: power requirement of 1 fan [W],
- LCOE: levelized cost of electricity [$ / kWh],
- $n_{\text{lifetime-years}}$: lifetime of fans in years,
- $c_{\text{fans}}^{\text{lifetime}}$: cost of fans over entire lifetime, and
- $c_{\text{HX}}^{\text{total}}$: total cost of entire heat exchanger over its lifetime.

The total cost comprises the cost of the air cooler itself and the lifetime cost of purchasing and running its fans. The cost of the cooler comprises the material cost of the finned tube (which comprises the cost of the tube and the fins and depends on the length of the tubes, the diameters of tube and fins and fin thickness) weighted to account for overhead related to construction and labour. The lifetime fan cost depends on the number of fans, their power requirement, the levelized cost of electricity, and the predicted lifetime.

Supplementary Text S4 The Simulator

Algorithm 1 1-D sCO_2 Heat-Exchanger Dynamic Length Simulation:

```
Input: T_c(0), T_h(0), P_c(0), P_h(0), T_h^target
n = 0
while T_h(n) > T_h^target do    ▷ If final segment temperature is too high increase the # of segments
    n = n + 1
    L = T_h(n−1) − 1
    U = T_h(n−1)
    T_h(n) = (L + U)/2
    T_h(n) → Compute Q_n, Q_htc
    while Q_n ≠ Q_htc do
        T_h(n) = BinarySearch(L, U)
        P_h(n) ← Compute pressure drop
        Q_n, Q_htc ← EnergyBalance(T_h(n), T_h(n−1), T_c(0), P_h(n), P_h(n−1))
    end while
    Q_CO2(n) ← HeatReleased(T_h(n), T_h(n−1), P_h(n), P_h(n−1))
    T_c(n) ← AirTemperature(Q_CO2(n), T_c(0))
end while
```

We present Algorithm 1 implemented for the heat exchanger dynamic length simulation.

To solve for temperatures and pressures at different points within the system, the simulator employs an iterative approach. First, it initializes a `Simulator` or `DynamicLength` instance with specified design parameters. Then, for each tube row in a sub-heat exchanger, it calls `_solve_tube()` method with initial conditions for CO_2 pressure (`p_co2_init`), temperature (`t_co2_init`), and optionally air inlet temperature(s) (`t_air_init`). This method solves for temperatures and pressures at each segment along a tube by calling `_solve_segment()` method iteratively.

In `_solve_segment()`, binary search is performed on output CO_2 temperature until the energy balance equation is satisfied within specified tolerance limits. For intermediate SHXs after the first one (`_intermediate_shx()`), binary search is performed on initial conditions until mean outlet temperature converges within tolerance limits. The calculated temperatures and pressures for each segment are stored in the results dictionary during the simulation

process.

The simulator uses physics-based equations such as energy balance equation to ensure that energy transferred from CO_2 to air through convection equals the energy change in CO_2 due to temperature variation and pressure drop. It also employs Log Mean Temperature Difference (LMTD) to calculate an average temperature difference between hot (CO_2) and cold (air) streams across a segment. Additionally, it computes an Overall Heat Transfer Coefficient based on tube geometry, fluid properties, and flow conditions. Finally, it estimates pressure drop across a segment using Darcy-Weisbach equation or other appropriate correlations.

By combining these physics equations with iterative solution techniques like binary search and segmented approach, this simulator can efficiently solve complex systems like multi-row heat exchangers with varying lengths or designs while maintaining accuracy in performance calculations.

Simulator

A computational approach to simulate temperature and pressure changes across a direct air-to-CO_2 heat exchanger/dry cooler, specifically in concentrated solar power applications, is replicated as per prior work.[S7,S8] This segment-by-segment approach, described in this entire section, allows for detailed adjustments of key design parameters to optimize performance, hence reduce the overall costs of producing and operating such a heat exchanger.

The heat exchanger is first split into two-dimensional segments perpendicular to the cross-section of the tubes. The heat transfer $Q_{i,j}$ in each segment (i, j) is then calculated using:

$$Q_{i,j} = O_{\mathrm{htc}(i,j)} \Delta \overline{T_{i,j}} \tag{S11}$$

$$= \dot{m}_c \left(T_{c(i,j+1)} - T_{c(i,j)} \right) \tag{S12}$$

$$= \dot{m}_h (T_{h(i,j)} - T_{h(i+1,j)}) \tag{S13}$$

where $\dot{m}_c$ and $\dot{m}_h$ are the mass flow rates of the air (cold fluid) and the sCO_2 (hot fluid) respectively, and where $\Delta\overline{T_{i,j}}$ is the log-mean temperature difference, defined by:

$$\Delta\overline{T_{i,j}} = \frac{\Delta T_{y_{i,j}} - \Delta T_{x_{i,j}}}{\ln \Delta T_{y_{i,j}} - \ln \Delta T_{x_{i,j}}} \tag{S14}$$

with $\Delta T_{y_{i,j}} = T_{h(i,j)} - T_{c(i,j+1)}$ and $\Delta T_{x_{i,j}} = T_{h(i+1,j)} - T_{c(i,j)}$ where T_h and T_c represent the temperatures of the hot and cold fluids respectively.

The overall heat transfer coefficient $O_{\text{htc}(i,j)}$ is then computed as follows:

$$O_{\text{htc}(i,j)} = \left[R_{\text{t}(i,j)} + R_{\text{a}(i,j)} + R_{\text{w}(i,j)}\right]^{-1} = \left[\frac{1}{h_{\text{s}}(A^{\text{s}}_{\text{sCO}_2})} + \frac{1}{h_{\text{air}}(A^{\text{s}}_{\text{air}})_{\text{eff}}} + 0\right]^{-1}_{(i,j)} \tag{S15}$$

where $R_{\text{t}(i,j)}$, $R_{\text{a}(i,j)}$, and $R_{\text{w}(i,j)}$ are the tube-side, the air-side, and the wall resistances, respectively at segment (i, j). Note that the wall resistances are assumed to be negligible in the analysis.

The air-side heat transfer cross-sectional and surface areas are given by Equations S16 and S17 respectively.[S7,S9]

$$A^{\text{c}}_{\text{air}} = (S_{\text{T}} - d^{\text{in}}_{\text{fin}})L_{\text{tube}} - (d^{\text{out}}_{\text{fin}} - d^{\text{in}}_{\text{fin}})t_{\text{fin}}n_{\text{fin}} \tag{S16}$$

$$(A^{s}_{\text{air}})_{\text{eff}} = \pi d^{\text{in}}_{\text{fin}}(L_{\text{tube}} - t_{\text{fin}}n_{\text{fin}}) + \pi n_{\text{fin}}\left(\frac{{d^{\text{out}}_{\text{fin}}}^2 - {d^{\text{in}}_{\text{fin}}}^2}{2} + d^{\text{out}}_{\text{fin}}t_{\text{fin}}\right)\eta_{\text{fin}} \tag{S17}$$

$$A^{s}_{\text{sCO}_2} = \pi d^{\text{in}}_{\text{tube}}L_{\text{tube}} \tag{S18}$$

$$\eta_{\text{fin}} = \frac{\tanh\left(\sqrt{\frac{2h_{\text{air}}}{k_{\text{f.m.}}t_{\text{fin}}}}\Phi\left(\frac{d^{\text{out}}_{\text{tube}}}{2}\right)\right)}{\sqrt{\frac{2h_{\text{air}}}{k_{\text{f.m.}}t_{\text{fin}}}}\Phi\left(\frac{d^{\text{out}}_{\text{tube}}}{2}\right)} \text{ where } \Phi = \left(\frac{d^{\text{out}}_{\text{fin}}}{d^{\text{out}}_{\text{tube}}} - 1\right)\left[1 + 0.35\ln\left(\frac{d^{\text{out}}_{\text{fin}}}{d^{\text{out}}_{\text{tube}}}\right)\right] \tag{S19}$$

where $A^{\text{c}}_{\text{air}}$: area of cross-section of air exposed to the flow, S_{T}: transverse pitch of tubes, $d^{\text{in}}_{\text{fin}}$: inside diameter of fins, $d^{\text{out}}_{\text{fin}}$: outside diameter of fins, t_{fin}: thickness of fins, n_{fin}: number of fins on each finned-tube, A^{s}_{air}: surface area of air exposed to heat exchange, i.e. surface area of tube that is in contact with air, L_{tube}: length of finned-tube, $d^{\text{in}}_{\text{tube}}$: inside diameter

of tubes, η_{fin}: fin efficiency, h_{air}: air-side heat transfer coefficient, $d_{\text{tube}}^{\text{out}}$: outside diameter of tubes, $k_{\text{f.m.}}$: thermal conductivity of fin material [W m^{-1} K^{-1}], and $A_{\text{sCO}_2}^{\text{s}}$: surface area of sCO_2 exposed to heat exchange, surface area of tube that is in contact with sCO_2.

The direct calculation of the outgoing thermodynamic properties in the heat exchanger is complicated due to the complex, non-linear, and non-differentiable factors involved in computing the overall heat transfer coefficient within each segment. Therefore, an iterative approach involving a binary search is used to solve for the outgoing sCO_2 temperature which satisfies Equations S11, S12 and S13. Note that this strategy is based on the assumption that the overall heat transfer coefficient, $O_{\text{htc}(i,j)}$, and by extension the heat transferred, is a monotonic function of outgoing sCO_2 temperature.

It is possible that, for a set of design parameters, a simulation run does not achieve the required outlet sCO_2 properties and to avoid this, the length of the tubes is dynamically adjusted to guarantee a successful design, possibly leading to higher overall cooler volumes and higher material costs. Should the volume be an important design constraint, the current procedure would need to be extended to accommodate this. Note that more detailed descriptions of the simulator are provided later on this section.

Using the same notation for geometrical variables as in Figure S1, the friction coefficients of airflow across finned tubes, $C_{f_{\text{air}}}$ are given below by Equations S20 and S21 for the simplified range of dimensions specified below:

$$C_{f_{\text{air}}} = 9.645 \cdot \text{Re}_{\text{max}}{}^{-0.316} \left(\frac{S_{\text{T}}}{d_{\text{tube}}^{\text{out}}} \right)^{-0.937} \tag{S20}$$

$$C_{f_{\text{air}}} = 3.805 \cdot \text{Re}_{\text{max}}{}^{-0.234} \left(\frac{s}{d_{\text{fin}}^{\text{out}}} \right)^{0.251} \left(\frac{s}{L_f} \right)^{-0.759} \left(\frac{d_{\text{fin}}^{\text{out}}}{d_{\text{tube}}^{\text{out}}} \right)^{-0.729} \left(\frac{d_{\text{tube}}^{\text{out}}}{S_{\text{T}}} \right)^{0.709} \left(\frac{S_{\text{L}}}{S_{\text{T}}} \right)^{-0.379} \tag{S21}$$

$$L_f = \frac{d_{\text{fin}}^{\text{out}} - d_{\text{fin}}^{\text{in}}}{2} \tag{S22}$$

where Re_{max}: Reynolds number defined with the outside diameter $d_{\text{tube}}^{\text{out}}$ and maximum velocity in the smallest cross-sectional area, and S_{L}: longitudinal pitch of tube arrangement (in Figure

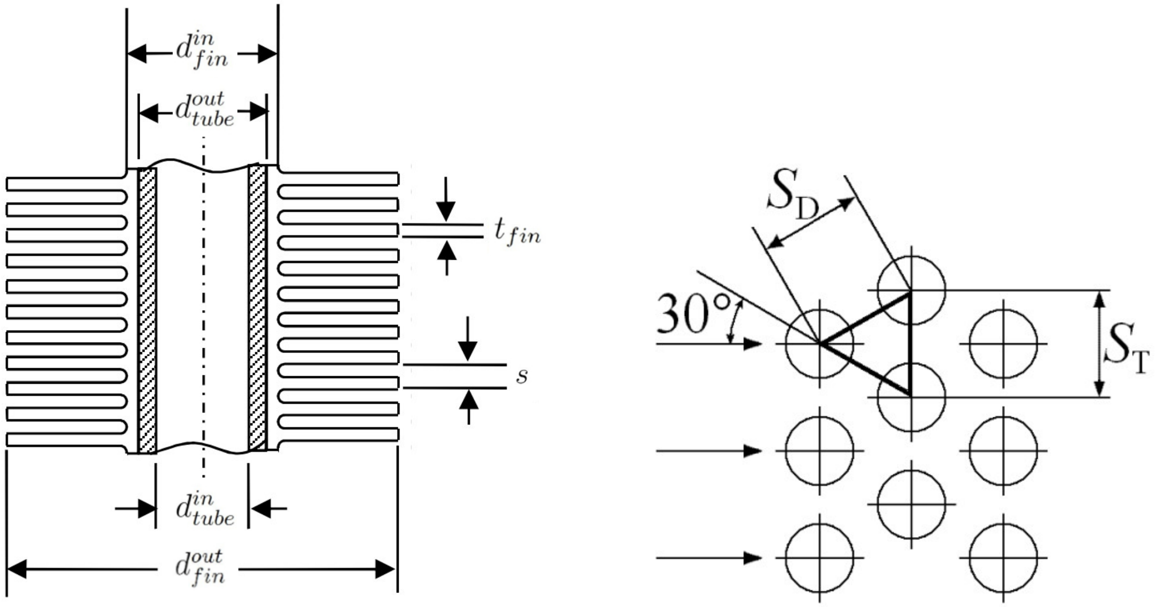

Figure S1: **Finned-tube dimensions and configuration. a)** Cross-section of finned-tube with dimensions.[S8] **b)** Tube bank with equilateral triangular arrangement: $S_T = S_D$.[S10]

S1b, using Pythagoras' Theorem: $S_D{}^2 = S_L{}^2 + (S_T/2)^2)$.

Note that the simplified range of dimensions used, which is a constrained form of the problem, is such that equation S20 is used when the fin size is greater than 0.0063m, and equation S21 is used otherwise. The full set of constraints under which these can be used is provided in prior work.[S10,S11]

These equations are then used to calculate the air pressure drop across the finned-tube.[S10,S11] Note that in this project, the air pressure drop, Δp_{air} is approximated by assuming that four rows of tubes are used with a configuration similar to Figure S1b for all designs.

$$\Delta p_{air} = \frac{G_{air}^2}{2\rho_{air}} \left(C_{f_{air}} \cdot 4 \cdot n_{rows}\right) \tag{S23}$$

where G_{air}: flow mass velocity of air [kg m^{-2}s^{-1}], ρ_{air}: density of air and n_{rows}: number of rows of tube per bundle.

This is then used to calculate the airflow for all the available fans which is in turn used to

calculate the number of fans required, n_{fans} as follows:

$$n_{\text{fans}} = \frac{\dot{m}_{\text{air}} \times 3000}{\text{airflow}_{\text{fanmodel}}} \tag{S24}$$

where $\dot{m}_{\text{air}}$: mass flow rate of air [kg s^{-1}], the factor of $\times 3000$ is for the conversion of the mass flow rate of air to [m^{-3}h^{-1}] and airflow$_{\text{fanmodel}}$: airflow of 1 fan of a specific model [m^{-3}h^{-1}].

This is then input into the cost calculator as described in Supplementary Text S3.

The air-side heat transfer coefficient, h_{air} is given by the following equation:[S7,S8,S12]

$$h_{\text{air}} = \frac{k_{\text{air}}}{d_{\text{tube}}^{\text{out}}} \left(0.134 \cdot \text{Pr}_{\text{air}}^{\frac{1}{3}} \text{Re}_{\text{air}}^{0.681} \left(\frac{2(s - t_{\text{fin}})}{d_{\text{fin}}^{\text{out}} - d_{\text{fin}}^{\text{in}}} \right)^{0.2} \left(\frac{s - t_{\text{fin}}}{t_{\text{fin}}} \right)^{0.1134} \right) \tag{S25}$$

where Re$_{\text{air}}$: Reynolds number of air, Pr$_{\text{air}}$: Prandtl number of air, k_{air}: thermal conductivity of air [W m^{-1} K^{-1}], $d_{\text{tube}}^{\text{out}}$: outside diameter of tube, s: fin pitch, t_{fin}: thickness of fins, $d_{\text{fin}}^{\text{out}}$: outside diameter of fin, and $d_{\text{fin}}^{\text{in}}$: inside diameter of fin.

To calculate the heat transfer coefficient of sCO_2, first calculate the pseudo-critical temperature of the fluid at a specified pressure P [bar] using the equation below:[S7,S13]

$$T_{pc} = 273.15 - 122.6 + 6.12P - 0.1657P^2 + 0.01773P^{2.5} - 0.0005608P^3 \tag{S26}$$

where T_{pc}: pseudo-critical temperature of sCO_2 [K] and P: pressure of sCO_2 [bar].
The sCO_2-side heat transfer coefficient, h_s is then given by the following equation:[S7,S8,S12,S14]

$$h_s = \frac{k_s \text{Nu}_s}{d_{\text{tube}}^{\text{in}}} = a \left(\text{Re}_s\right)^b \left(\text{Pr}_s\right)^c \left(\frac{\rho_{pc}}{\rho_s} \right)^n \left(\frac{k_s}{d_{\text{tube}}^{\text{in}}} \right) \tag{S27}$$

$$\text{When } T_s > T_{pc} : a = 0.14,\ b = 0.69,\ c = 0.66,\ n = 0 \tag{S27a}$$

$$\text{When } T_s \leq T_{pc} : a = 0.013,\ b = 1.0,\ c = -0.05,\ n = 1.6 \tag{S27b}$$

where Re$_s$: Reynolds number of sCO_2, Pr$_s$: Prandtl number of sCO_2, Nu$_s$: Nusselt number of the sCO_2, k_s: thermal conductivity of sCO_2 [W m^{-1} K^{-1}], ρ_{pc}: density of sCO_2 at

pseudo-critical point [kg m^{-3}], and ρ_s: density of sCO_2 [kg m^{-3}].

The following equations are used to calculate the sCO_2 pressure drop, Δp_s along a tube segment, L_{segment}:[S7,S8,S12]

$$C_{f_s} = 8\left[\left(\frac{8}{\text{Re}_s}\right)^{12} + \left\{2.457\left[\ln\left(\frac{1}{\left(\frac{7}{\text{Re}_s}\right)^{0.9} + 0.27\left(\frac{\epsilon}{d_{\text{tube}}^{\text{in}}}\right)}\right)\right]^{16} + \left(\frac{37530}{\text{Re}_s}\right)^{16}\right\}^{-\frac{3}{2}}\right]^{\frac{1}{12}} \tag{S28}$$

$$u_s = \frac{\dot{m}_s}{\rho_s \frac{\pi\left(d_{\text{tube}}^{\text{in}}\right)^2}{4}} = \frac{4\dot{m}_s}{\rho_s \pi \left(d_{\text{tube}}^{\text{in}}\right)^2} \tag{S29}$$

$$\Delta p_{\text{s}} = \frac{\rho_{\text{s}} C_{f_{\text{s}}} \left(u_s\right)^2}{2 d_{\text{tube}}^{\text{in}}} L_{\text{segment}} \tag{S30}$$

where ϵ: relative surface roughness of tube, $d_{\text{tube}}^{\text{in}}$: inside diameter of tube, and Re_s: Reynolds number of sCO_2, u_s: flow velocity of sCO_2 [m s^{-1}], ρ_s: density of sCO_2 [kg m^{-3}], $\dot{m}_s$: mass flow rate of sCO_2 [kg s^{-1}], C_{f_s}: friction coefficient of sCO_2 through the tube, and L_{segment}: length of tube segment.

Dynamic Length Simulator.

The Dynamic Length Simulator (DLS) is designed to optimize the length of tubes in a system by incrementally increasing the number of tube segments until a target temperature is reached. The DLS calculates relevant temperatures along each row, one tube at a time, and employs an energy balance approach to ensure accurate results.

Simulation Process

Note that this part of the simulation involves using CoolProp. It is an open-source database of fluid and humid air properties, formulated based on the most accurate formulations in open literature. It has been validated against the most accurate data available from the relevant references.[S15] The simulation is described in the following series of steps:

1. Initialization: The simulation begins by recovering the output air temperature along the length of the previous tube segment

2. Dynamic Scaling: Using dynamically scaled temperature bounds, a binary search over output sCO_2 temperatures is performed for each segment

3. Downstream Propagation: The solver progresses downstream from the first row of tubes that meets a roughly uniform air temperature, and continues downstream for the remaining rows of tubes where each segment encounters monotonically decreasing air temperatures.

4. Energy Balance Propagation: Starting with the inlet sCO_2 temperature, the solver propagates energy balance downstream of the sCO_2 flow along each tube segment. The number of these segments is increased until the average outlet sCO_2 temperature across all tubes (in the first row in contact with the air inlet) reaches the target sCO_2 outlet temperature.

Energy Balance Calculation.

It is vital that energy conservation is satisfied throughout the simulation process. This is done by calculating the required energy balances in the following steps:

1. Calculate the energy lost by sCO_2 using both guessed output sCO_2 temperature and known input values for air and input sCO_2 temperatures (Equation S13)

2. Determine output air temperature based on energy balance between air and sCO_2 (Equation S12): If air is too hot (hotter than input/output sCO_2), re-evaluate with a higher output sCO_2 temperature.

3. Compute the overall heat transfer (Q_{oht}) from the tube segment at given air and sCO_2 temperatures and pressures using Equation S11. Then:

- If $Q_{\mathrm{oht}} \approx Q_{\mathrm{sCO_2}}$: Done
- If $Q_{\mathrm{oht}} > Q_{\mathrm{sCO_2}}$: Decrease output sCO_2 temperature due to insufficient heat radiation from sCO_2
- If $Q_{\mathrm{oht}} < Q_{\mathrm{sCO_2}}$: Increase output sCO_2 temperature due to excessive heat radiation from CO_2

Supplementary Text S5 Expanded Optimization Details

The time taken to select a new design by the optimizer and the time taken per simulation is also monitored and shown in Figure S2. Note that within the optimization loop, the simulator has to be run to evaluate candidates for the new designs, and time to get new set of design parameters excludes this.

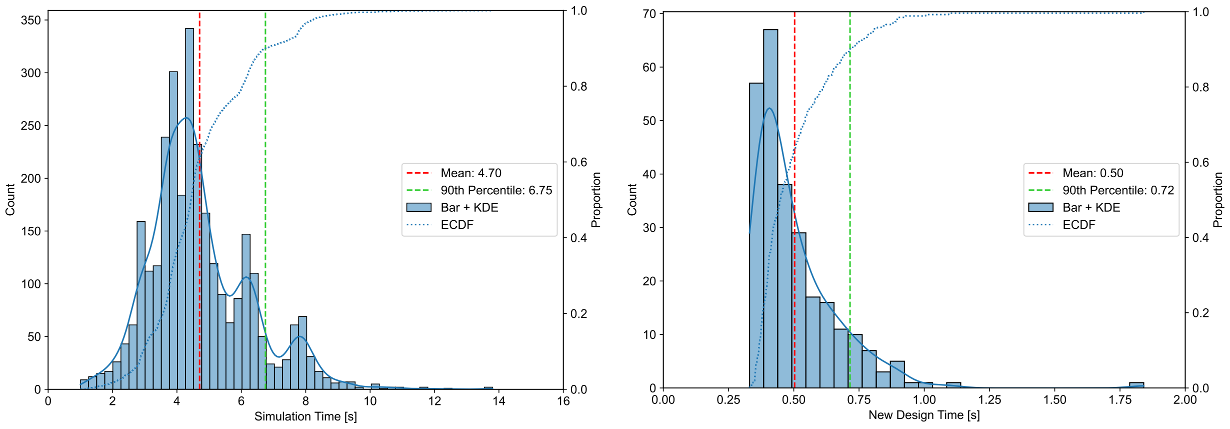

Figure S2: **Monitoring of time taken for simulations and generating new sets of design parameters.** Note that this excludes any simulations performed within the optimization step itself, i.e., for initialization of candidate sets of parameters. Here, the total number of simulations is 3000 and the number of new design choices by the optimizer is 264. **a)** The time for each simulation performed at each iteration. **b)** The time for the optimizer to select a new set of design parameters.

Supplementary Text S6 Model Parameters and Bounds

Tables S1 and S2 show the constant input parameters of the 25 MW CSP plant and the reference design parameters of the finned-tubes being considered in this project. These are based on prior work.[S7,S8] Moreover, the CSP operating conditions kept constant throughout the experiments are shown in Table S3 (based on[S8]) while the parameter optimization bounds and cost parameters being applied in this project are provided in Tables S4 and S5 respectively. Note that in Table S4, the upper bound for ΔT_{air} is dynamically calculated using the following equation:

$$(\Delta T_{\text{air}})_{\text{ub}} = \eta_{\text{HX}} \frac{\dot{m}_s (c_p)_s (T_s^{\text{in}} - T_s^{\text{out}})}{\dot{m}_{\text{air}} (c_p)_{\text{air}}} \tag{S31}$$

where η_{HX}: overall efficiency of the heat exchanger, $\dot{m}_s$: mass flow rate of sCO_2 [kg s^{-1}], $\dot{m}_{\text{air}}$: mass flow rate of air [kg s^{-1}], $(c_p)_s$: specific heat capacity of sCO_2 [J kg^{-1} K^{-1}], $(c_p)_{\text{air}}$: specific heat capacity of air [J kg^{-1} K^{-1}], T_s^{in}: inlet temperature of sCO_2, and T_s^{out}: outlet temperature of sCO_2.

Table S1: Tube-side input parameters.

**Tube-side specification**		
**Parameter**	**Value**	**Unit**
Tube material	ASTM A214 mild steel	-
Tube material density, $\rho_{\text{t.m.}}$	7950	kg m^{-3}
Thermal conductivity of tube material, $k_{\text{t.m.}}$	50	W m^{-1} K^{-1}
Tube outside diameter, $d_{\text{tube}}^{\text{out}}$	25	mm
Tube inside diameter, $d_{\text{tube}}^{\text{in}}$	20	mm
Tube segment length, L_{segment}	200	mm
Relative tube surface roughness, $\epsilon/d_{\text{tube}}^{\text{in}}$	5.24×10^{-4}	-
Number of tube rows, n_{rows}	4	-
Effective no. of tubes per row, $n_{\text{tubes-in-row}}$	47.5	-
No. of tubes per bundle, n_{bundles}	190	-
Transversal tube pitch, S_{T}	58	mm
Longitudinal tube pitch, S_{L}	52	mm

Table S2: Fin-side input parameters.

Fin-side specification		
**Parameter**	**Value**	**Unit**
Fin type	Extruded bimetallic	-
Heat exchanger arrangement	Horizontal	-
Fin shape (in direction of sCO2 flow)	Circular	-
Tube arrangement	Staggered	-
Fin material	ASTM 6063 aluminium	-
Fin material density, $\rho_{\text{f.m.}}$	2750	kg m^{-3}
Thermal conductivity of fin material, $k_{\text{f.m.}}$	204	W m^{-1} K^{-1}
Fin outer diameter, $d_{\text{fin}}^{\text{out}}$	57	mm
Fin inner diameter, $d_{\text{fin}}^{\text{in}}$	28	mm
Fin shape (in thickness terms)	Uniform	-
Fin thickness, t_{fin}	0.5	mm
Fin Pitch, s	2.8	mm

Table S3: Operating conditions of CSP model held constant throughout the experiments.

CSP operating conditions		
**Parameter**	**Value**	**Unit**
Inlet atmospheric pressure at ground level, $p_{\text{air}}^{\text{in}}$	99695	N m^{-2}
Condition of air	Dry	–
sCO_2 inlet temperature, T_s^{in}	71	°C
sCO_2 mean outlet temperature (first row), T_s^{out}	40.3	°C
sCO_2 inlet pressure, p_s^{in}	7.5	MPa
sCO_2 mass flow rate, $\dot{m}_s$	406.6	kg s^{-1}
Universal gas constant, R	287.08	J kg^{-1} K^{-1}
Heat exchanger efficiency, η_{HX}	0.6	-

Table S4: Parameter bounds applied to the optimization of the CSP model to achieve feasible designs from a physical and engineering perspective.

CSP model optimization bounds			
**Parameter**	**Lower Bound**	**Upper Bound**	**Unit**
$d_{\text{tube}}^{\text{in}}$	10	40	mm
Ratio $d_{\text{tube}}^{\text{out}}/d_{\text{tube}}^{\text{in}}$	1.1	2	-
Ratio $d_{\text{fin}}^{\text{in}}/d_{\text{tube}}^{\text{out}}$	11.13/11	2	-
Ratio $d_{\text{fin}}^{\text{out}}/d_{\text{fin}}^{\text{in}}$	13.97/11.13	2.1	-
Ratio $S_{\text{T}}/d_{\text{fin}}^{\text{out}}$	1.01	2.1	-
Fin pitch, s	1	4	mm
Ratio t_{fin}/s	0.1	0.8	-
ΔT_{air}	1	$(\Delta T_{\text{air}})_{\text{ub}}$	°C

Table S5: Cost calculations parameters.

**Cost calculations parameters**		
**Parameter**	**Value**	**Unit**
Cost of fin material, $c_{\mathrm{f.m.}}$	4.2	\$ / kg
Cost of tube material, $c_{\mathrm{t.m.}}$	0.8	\$ / kg
Fixed cost of finned-tubes per unit length, $c^{\mathrm{fixed}}_{\mathrm{finned-tube}}/L$	2	\$ / m
Weighting factor per unit length, $f_{\mathrm{weighting}}$	2	m^{-1}
Weighting factor for heat exchanger header cost, f_{header}	0.8	-
Weighting factor for heat exchanger labor cost, f_{labour}	0.7	-
Weighting factor for heat exchanger, f_{HX}	1.2	-
Operating lifetime of heat exchanger, $n_{\mathrm{lifetime-years}}$	25	years
Assumed cost of fan power, LCOE	0.05	\$ / kWh

References

(S1) Weller, H.; Tabor, G.; Jasak, H.; Fureby, C. A Tensorial Approach to Computational Continuum Mechanics Using Object Orientated Techniques. *Computers in Physics* **1998**, *12*, 620–631.

(S2) Sengupta, M.; Xie, Y.; Lopez, A.; Habte, A.; Maclaurin, G.; Shelby, J. The National Solar Radiation Data Base (NSRDB). *Renewable and Sustainable Energy Reviews* **2018**, *89*, 51–60.

(S3) Wikipedia Comma-separated values - Wikipedia. `https://en.wikipedia.org/wiki/Comma-separated_values`, 2022; [Online; accessed 07-July-2023].

(S4) Kröger, D. *Air-cooled Heat Exchangers and Cooling Towers*; Penwell Corporation, 2004; Vol. 1.

(S5) Kröger, D. *Air-cooled Heat Exchangers and Cooling Towers*; Penwell Corporation, 2004; Vol. 2.

(S6) Ehsan, M. M.; Guan, Z.; Gurgenci, H.; Klimenko, A. Feasibility of dry cooling in supercritical CO2 power cycle in concentrated solar power application: Review and a case study. *Renewable and Sustainable Energy Reviews* **2020**, *132*, 110055.

(S7) Khatoon, S.; Ishaque, S.; Kim, M.-H. Modeling and analysis of air-cooled heat exchanger integrated with supercritical carbon dioxide recompression Brayton cycle. *Energy Conversion and Management* **2021**, *232*, 113895.

(S8) Ehsan, M. M.; Wang, X.; Guan, Z.; Klimenko, A. Design and performance study of dry cooling system for 25 MW solar power plant operated with supercritical CO2 cycle. *International Journal of Thermal Sciences* **2018**, *132*, 398–410.

(S9) Monjurul Ehsan, M.; Guan, Z.; Klimenko, A.; Wang, X. Design and comparison

of direct and indirect cooling system for 25 MW solar power plant operated with supercritical CO_2 cycle. *Energy Conversion and Management* **2018**, *168*, 611–628.

(S10) Saari, J. Heat Exchanger Thermal Design Guide. 2010.

(S11) Rohsenow, W.; Hartnett, J.; Ganić, E. *Handbook of Heat Transfer Applications*; Mechanical engineering; McGraw-Hill, 1985; pp 4–237.

(S12) Yoon, S. H.; Kim, J. H.; Hwang, Y. W.; Kim, M. S.; Min, K.; Kim, Y. Heat transfer and pressure drop characteristics during the in-tube cooling process of carbon dioxide in the supercritical region. *International Journal of Refrigeration* **2003**, *26*, 857–864.

(S13) Liao, S.; Zhao, T. Measurements of Heat Transfer Coefficients From Supercritical Carbon Dioxide Flowing in Horizontal Mini/Micro Channels. *Journal of Heat Transfer-transactions of The Asme - J HEAT TRANSFER* **2002**, *124*.

(S14) Ehsan, M. M.; Duniam, S.; Li, J.; Guan, Z.; Gurgenci, H.; Klimenko, A. Effect of cooling system design on the performance of the recompression CO2 cycle for concentrated solar power application. *Energy* **2019**, *180*, 480–494.

(S15) Bell, I. H.; Wronski, J.; Quoilin, S.; Lemort, V. Pure and Pseudo-pure Fluid Thermophysical Property Evaluation and the Open-Source Thermophysical Property Library CoolProp. *Industrial & Engineering Chemistry Research* **2014**, *53*, 2498–2508.
